# Pediatric Melioidosis in Central India: A Clinico-Epidemiological Study From a Landlocked Region

**DOI:** 10.7759/cureus.98488

**Published:** 2025-12-04

**Authors:** Malti Dadheech, Ayush Gupta, Deshanshi Richariya, Yogendra S Yadav

**Affiliations:** 1 Department of Microbiology, All India Institute of Medical Sciences, Bhopal, Bhopal, IND; 2 Department of Paediatrics, All India Institute of Medical Sciences, Bhopal, Bhopal, IND

**Keywords:** burkholderia pseudomallei, central india, clinical spectrum, emerging infectious disease, madhya pradesh, melioidosis, pediatric infection

## Abstract

Objective

Melioidosis, caused by *Burkholderia pseudomallei*, is an emerging infectious disease in India but remains less common in children compared with adults. Most studies are reported from southern and eastern coastal states, with limited data from central India, a landlocked, traditionally non-endemic region.

Materials and methods

We conducted a part-retrospective, part-prospective study at a tertiary care institute in central India between January 2020 and September 2025. The study included all patients aged ≤16 years with isolation of *B. pseudomallei* in clinical samples. The clinico-epidemiological and laboratory data were obtained from medical records, and patients were followed up prospectively to know the outcome. Identification was performed using Vitek^®^-2 Compact till 2022 and later all the isolates were confirmed by Vitek^®^ MS Prime (both bioMérieux, Marcy-l'Étoile, France). Antimicrobial susceptibility was determined by the Kirby-Bauer method following European Committee on Antimicrobial Susceptibility Testing (EUCAST) guidelines.

Results

Among 132 confirmed melioidosis cases, a total ofseven (5%) pediatric melioidosis cases were identified over a six-year period. The most common clinical presentation was pneumonia in three (42%), visceral organ abscess in two (28%), soft tissue abscesses in two (28%) followed by lymphadenitis in one (14%) and neurological involvement in one (14%) case. Two cases had multisystemic involvement. None of the patients had diabetes, while three (42%) had other comorbidities. Two (28%) children succumbed despite no known comorbidities, while the remaining improved with standard treatment for melioidosis.

Conclusion

Melioidosis presents with varied clinical manifestations in pediatric patients and should be considered in the differential diagnosis in patients with pneumonia, visceral abscesses and lymphadenitis, even in the absence of traditional risk factors. The emergence of pediatric cases in a non-coastal, central Indian region indicates a shift in epidemiological pattern and highlights the need for increased clinical vigilance. Early diagnosis and timely treatment remain essential to reduce mortality.

## Introduction

Melioidosis, a tropical and sub-tropical disease, is caused by the sapronotic bacterium Burkholderia pseudomallei (Bpm). It is transmitted through inhalation of contaminated dust, inoculation into the skin, or possibly by ingestion of contaminated water [1]. According to a modelling study, the highest predicted incidence is in South Asia, particularly in India, which bears the highest population burden [2]. Within India, the southern and eastern coastal states, particularly Karnataka, Tamil Nadu, Kerala, and Odisha, are considered a hotspot [3,4]. Despite this, the disease remains grossly underdiagnosed in other parts of the country [5].

Melioidosis has diverse clinical manifestations ranging from seemingly innocuous skin and soft tissue infections to more serious pneumonia, deep organ abscesses often leading to multisystemic involvement with septic shock [1]. This protean clinical picture makes early diagnosis challenging, leading to the high mortality rate between 10-50% [1,6]. In adults, the disease is largely associated with certain predisposing risk factors such as diabetes mellitus, chronic kidney or lung disease, and long-term alcoholism [1].

The published literature is limited on pediatric melioidosis when compared to adults, with the pediatric population accounting for only 5-15% of total cases in endemic regions [7]. Unlike adults, cases in children mostly occur without predisposing factors [8]. Published literature suggests that children are less likely to develop disseminated disease, however mortality typically occurs in those with bacteremia and disseminated disease [7]. The literature suggests that the clinical presentation of the pediatric population differs from adults [8]. While pneumonia is commonly reported in adults, children often present with localized infection in the form of cutaneous infection, suppurative lymphadenitis, or parotitis [7,8].

Regional variations in pediatric disease burden and manifestations have also been observed globally [7]. In India, reports of pediatric melioidosis remain sparse and fragmented, with very few cases documented in the literature [9-15]. We have been regularly diagnosing cases of melioidosis at our institute, situated in central India, since 2020. We conducted a review of diagnosed cases at our institute to delineate the clinico-epidemiological spectrum of pediatric melioidosis.

## Materials and methods

We conducted this part-retrospective, part-prospective study at an academic, tertiary care institute of national importance in central India. This design was adopted to capture and report all culture-confirmed pediatric melioidosis cases diagnosed over six years. Cases diagnosed between January 2020 and December 2022 were included retrospectively based on medical records, while those identified between January 2023 and September 2025 were enrolled prospectively as part of an ongoing research project initiated at our center.

The case definition included all patients aged ≤16 years, presenting to our center between January 2020 and September 2025 with isolation of Bpm in a clinical sample. Clinical syndromes were further categorized as acute (symptoms present for less than two months) and chronic (symptoms present for two or more months) presentation [16]. The clinico-epidemiological details, laboratory findings, and case management were collected using medical records, and patients were followed up prospectively to know the outcome. Ethical approval was obtained from the institute’s institutional human ethics committee for retrospective analysis (vide No.-ihecsr/aiimsbpl/aug/84) and for prospective enrolment (vide No. IHEC-LOP/2024/EL037).

Prior to 2023, clinical samples were processed on standard culture media (Himedia, Mumbai, India), for aerobic culture and sensitivity testing such as blood agar, chocolate agar and MacConkey agar, as applicable. Since 2023, the pus and respiratory clinical samples from suspected cases were also inoculated on selective culture media for Bpm such as crystal violet colistin-50 (CVC-50) enrichment broth and Ashdown agar (AA). The CVC-50 broth and AA were incubated for five days before being declared negative for Bpm. Suspected colonies were screened using Gram-negative reaction, oxidase positivity, and triple disc test, i.e., resistance to colistin (10mg) and gentamicin (10mg) and susceptibility to amox-clavulanate (20/10mg) disk on Mueller-Hinton agar (Himedia) disk diffusion plate. Identification was performed using Vitek®-2 Compact (bioMérieux, Marcy-l'Étoile, France) till 2022 and later all the isolates were confirmed by Vitek®-MS Prime database version 3.2 (bioMérieux). Antibiotic susceptibility was determined using the Kirby-Bauer disk diffusion method, interpreted according to European Committee on Antimicrobial Susceptibility Testing (EUCAST) guidelines [17]. The anti-Bpm antibiotics tested were amox-clavulanate (20-10mg), ceftazidime (10mg), imipenem (10mg), meropenem (10mg), co-trimoxazole (1.25-23.75mg), chloramphenicol (30mg) and tetracycline (30mg) as a surrogate for doxycycline.

## Results

A total of 132 culture-confirmed melioidosis cases were diagnosed at our centre between 2020-2025, of which seven (5%) cases were documented in pediatric and adolescent patients (≤16 years). The median age of presentation was five years (range, 2-16) with a male predominance (male:female ratio, 6:1). Individual case-wise clinical presentations, epidemiological factors, diagnosis, and management are described below and summarised in Table 1.

**Table 1:** Clinico-epidemiological profile, laboratory characteristics, and outcome of pediatric melioidosis cases Abbreviations:  F, female; M, male; AFI, acute febrile illness; CAP, community acquired pneumonia; DSA, deep seated abscess; SSTI, skin and soft tissue infection; LRTI, lower respiratory tract infection; USG, ultrasonography; CECT, contrast-enhanced computed tomography; HRCT, high-resolution computed tomography; MRI, magnetic resonance imaging; iv, intravenous; po, per oral

Case details	Case 1	Case 2	Case 3	Case 4	Case 5	Case 6	Case 7
Age/Gender	4/F	5/M	2/M	11/M	16/M	4/M	16/M
Month/Year of presentation	August/2022	October/2022	September/2023	August/2024	October/2024	December/2024	August/2025
Social background	Urban	Rural	Rural	Rural	Rural	Urban	Urban
Comorbidity	None	None	Sickle cell disease	None	Chronic kidney disease	Spina bifida, Neurofibromatosis type1	None
Presentation	Acute	Chronic	Acute	Acute	Acute	Acute	Acute
Clinical Syndrome	AFI with CAP	DSA (Liver)	AFI with CAP, SSTI	Cervical Lymphadenitis	AFI with LRTI, DSA (Liver, Kidney)	SSTI	DSA (Brain)
Key imaging findings	Bilateral lung infiltrates (Chest X-ray)	Multiple liver abscesses (USG/ CECT abdomen)	Bilateral cavitary lesions with consolidatory nodules (HRCT Thorax)	Not done	Cavitary lesions with consolidation and nodules (HRCT Thorax)	Not done	Solitary Brain abscess in fronto-parietal lobe (MRI/CECT Brain)
Organs involved	Lungs	Liver	Lungs, Skin	Lymph node	Lungs, Liver, Kidney	Soft tissue (Thigh)	Brain
Positive culture specimen	Blood	Blood, Pus (Liver)	Pus (forearm)	Pus (Lymph node)	Broncho-alveolar lavage	Pus from external fixator site	Pus (Brain)
In-hospital outcome	Death	Discharged	Discharged	Discharged	Discharged	Discharged	Death
In-patient treatment	iv meropenem	iv ceftazidime	iv meropenem, po co-trimoxazole	po amox-clavulanate	Managed through outpatient	iv meropenem, iv ceftazidime	iv meropenem
Treatment after discharge	NA	po co-trimoxazole	po co-trimoxazole	po amox-clavulanate	iv ceftazidime po co-trimoxazole	po co-trimoxazole	NA
Follow up	NA	Alive	Alive	Alive	Alive	Alive	NA

Case 1: Acute febrile illness with septic shock

**Figure 1:**
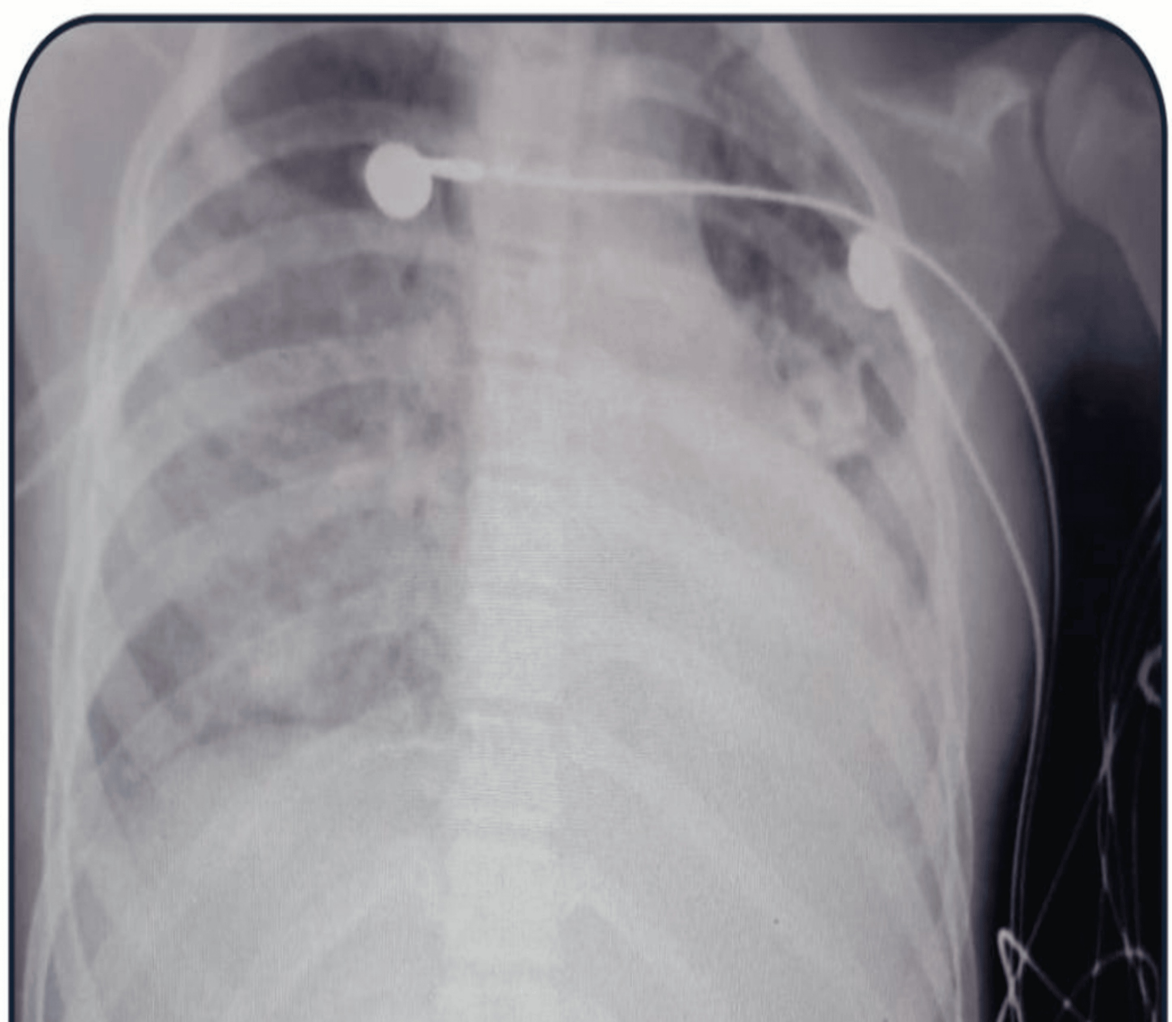
Chest radiograph showing generalized bilateral lung infiltrates

A four-year-old, previously healthy girl, presented in the pediatric emergency with an acute onset of high-grade fever for five days, loose stools with vomiting, and abdominal pain for one day. On admission, the patient was disoriented, Glasgow Coma Score (GCS) was E4V4M5, non-recordable blood pressure, petechial rash around the lips, and conjunctival congestion. Heart rate was 170 beats per minute (bpm), respiratory rate was 43/minute, and extremities were cold, suggesting severe shock. A presumptive diagnosis of acute gastroenteritis with shock and severe metabolic acidosis was made. Patient was immediately admitted to the intensive care unit (ICU) and given immediate airway management, intravenous (iv) fluids, and inotropes. Blood samples for routine hemogram, liver function test (LFT), renal function test (RFT), and blood culture (PF Plusâ, BacT/Alert 3D, bioMérieux) were collected before initiating the patient on iv meropenem and vancomycin. Initial hemogram showed normal haemoglobin (11.1 mg/dl), leukopenia (2.06/mm3 and severe thrombocytopenia (46000/ml) while LFT and RFT showed mild hyperbilirubinemia (2.52 mg/dL) and raised urea (107.75 mg/dL) and creatinine (3.04 mg/dL). Chest X-ray revealed generalized bilateral lung infiltrates (Figure 1).

Within six hours of admission, patient suffered cardiac arrest and could not be revived. One day later, the blood culture bottle flagged positive showing Gram-negative bacilli which was later identified to be Bpm susceptible to standard anti-Bpm antimicrobials.

Case 2: Liver abscess

**Figure 2:**
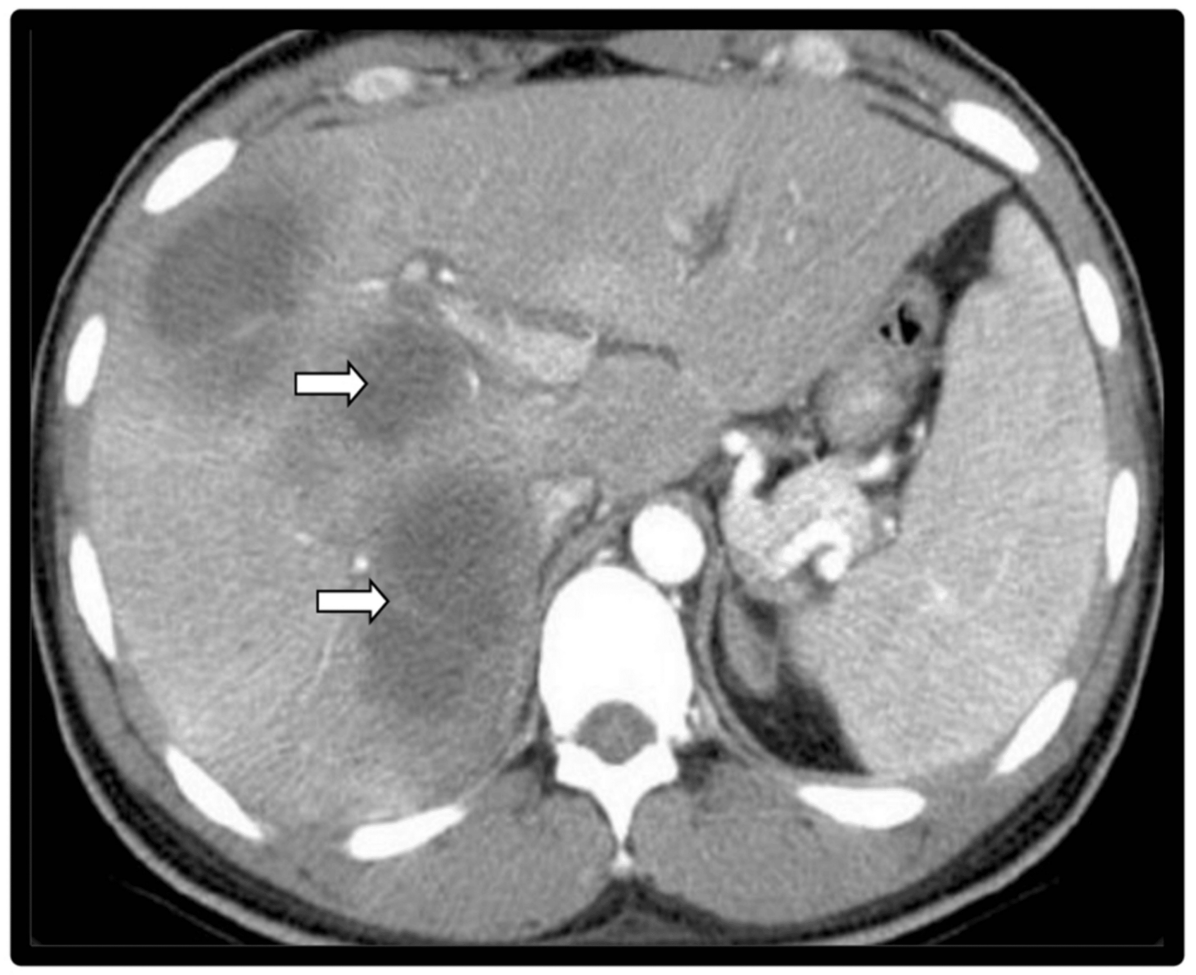
Contrast-enhanced computed tomography (CECT) (abdomen) image showing multiple liver abscesses

A five-year-old malnourished boy presented in pediatric emergency with complaints of gradually progressive high-grade fever, right hypochondriac pain, and abdominal distension for 15 days. Patient’s initial condition was stable with normal blood pressure, mild tachycardia, and tachypnoea. Per-abdomen examination showed abdominal distension with shifting dullness. The patient was previously hospitalized around one month back in the Pediatric Surgery department with complaints of on-and-off fever of two months duration with multiple liver abscesses on ultrasound, for which USG-guided drainage was done. A presumptive diagnosis of ruptured liver abscess was made, and the patient was started on iv piperacillin-tazobactam and metronidazole after collecting the blood culture sample. On admission, the patient was having severe anemia (Hb: 3.2 g/dL) and leukocytosis (20.06/mm3). Both USG and contrast enhanced computed tomography (CECT) abdomen showed multiple hypodense lesions in both lobes of the liver, with the largest measuring 2.9 X 3.1 X 3.1 cm, with a few lesions (Figure 2).

A plan of diagnostic pus aspiration under imaging guidance was made. Both blood culture and aspirated pus from the liver grew B. pseudomallei susceptible to anti-Bpm drugs. After culture reports, the patient was shifted to iv ceftazidime, which was continued for three weeks, leading to clinical improvement. Repeat blood culture was sterile, and follow-up USG showed a reduction in abscess size. Patient was discharged on oral co-trimoxazole for three months along with amoxicillin-clavulanic acid for three weeks. Upon telephonic follow-up after two years, the patient was doing fine and didn’t relapse.

Case 3: Community-acquired pneumonia (CAP) with cutaneous manifestations

**Figure 3:**
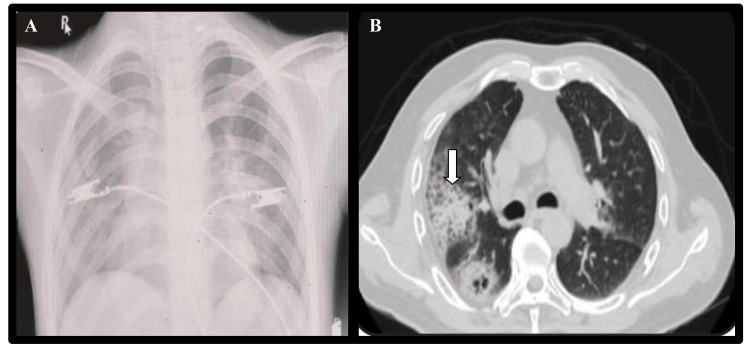
(A) Chest radiograph showing multiple bronchoalveolar opacities with diffuse haziness (black arrow) (B) High-resolution computed tomography (HRCT) chest showing necrotizing/cavitating pneumonia (white arrow)

A two-year-old boy, a known case of sickle cell disease, presented in the emergency with fever for two months, cough for seven days, and recurrent pus discharge from a wound over the scalp. Two months earlier, he had sustained a traumatic laceration to the scalp after falling in a muddy area, which was immediately sutured. However, the wound became infected, requiring incision and drainage at a local hospital. Subsequently, the patient developed purulent swellings on the forearm and lower back, with continuing fever, after which he was referred to our centre. On admission, the initial CXR showed multiple bronchoalveolar opacities with diffuse haziness (Figure 3A). High-resolution computed tomography (HRCT) Thorax showed multiple consolidatory nodules and cavitary lesions scattered in bilateral lung parenchyma with air bronchogram sign in the right lower lung, suggestive of necrotizing/cavitating pneumonia (Figure 3B).

Initially, the patient was given iv piperacillin-tazobactam and vancomycin in view of multiple skin abscesses and pneumonia, and was worked up for chronic granulomatous disease, but results were negative. Surgical drainage of abscesses was done, and pus culture grew Bpm, while blood cultures were repeatedly sterile. After establishing the etiological diagnosis, antibiotics were changed to iv meropenem and oral co-trimoxazole for 10 days. There was resolution of the fever and regression of the abscess after specific antimicrobials were given. The patient was subsequently discharged on oral co-trimoxazole for six months and has remained asymptomatic till now.

Case 4: Acute suppurative lymphadenitis

**Figure 4:**
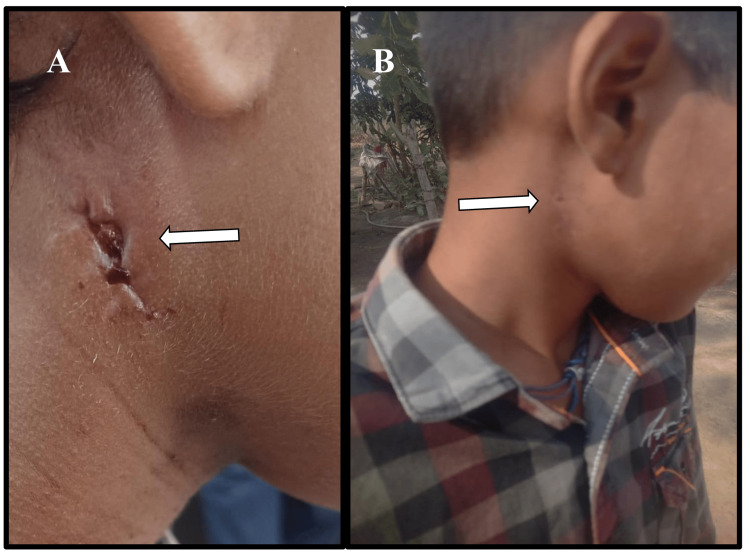
Clinical images of pediatric melioidosis case (A) Ulcerative cutaneous lesion on the right side of the neck (white arrow) (B) Healed lesion site following treatment (white arrow)

An 11-year-old boy presented in the outpatient department (OPD) of the Otolaryngology department, with a gradually progressive painful swelling on the right side of the neck for one month. It was associated with evening rise of fever, cough, and weight loss over three months. Examination revealed a tender, fluctuant 3X3 cm in size at the right level-II lymph node (Figure 4A). On examination, no other systemic abnormalities were noted. With a suspicion of tubercular lymphadenitis, the patient was admitted for incision and drainage, and pus was sent for bacterial culture, mycobacterial PCR, acid-fast staining, and histopathological evaluation. The patient was empirically treated with oral amoxicillin-clavulanate and subsequently discharged. The pus culture grew Bpm, and was negative for investigations pertaining to mycobacterial detection. The patient was reviewed in OPD, where amoxicillin-clavulanate was continued for three months. On follow-up, the patient has recovered completely (Figure 4B).

Case 5: Lower respiratory tract infection

**Figure 5:**
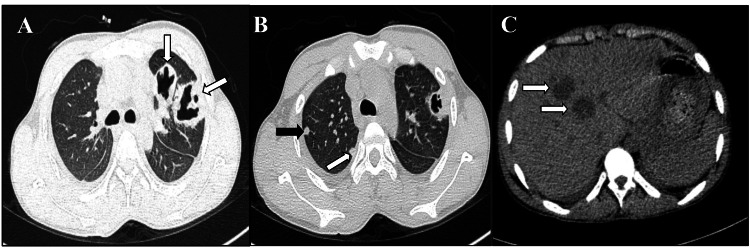
High-resolution computed tomography (HRCT) thorax (axial lung window) showing (A) areas of cavitatory consolidation in left upper lobe (white arrows) (B) few random and subpleural nodules in right upper lobe (white and black arrows) (C) Contrast-enhanced computed tomography (CECT) (upper abdomen) showing well defined homogeneous hypodense lesions in liver (white arrows)

A 16-year-old male, known case of chronic kidney disease secondary to obstructive uropathy, presented in the emergency with complaints of abdominal pain, dyspnea, and fever for three days. Investigations revealed neutrophilic leukocytosis (2.46/mm3), severe anemia (Hb: 5.9 mg/dL), and raised serum creatinine (10.4 mg/dL). Urine routine microscopy was suggestive of urinary tract infection. He was managed with IV antibiotics, supportive measures, and one unit of packed red blood cell transfusion, following which renal function improved. Due to persistent cough, pleuritic chest pain, and fever, HRCT thorax and CECT abdomen were performed. HRCT thorax showed areas of cavitatory consolidation in left upper lobe and few random and subpleural nodules in right upper lobe (Figure 5A, 5B), CECT abdomen showed well-defined homogeneous hypodense lesions in liver (Figure 5C) for which he was advised to undergo diagnostic bronchoscopy. In addition, there were multiple hepatic and renal abscesses.

Broncho-alveolar lavage sample was collected a week later and sent for investigations pertaining to aerobic bacterial, mycobacterial, and fungal etiologies. The aerobic bacterial culture grew Bpm as well. The patient was given specific anti-Bpm treatment and has improved clinically.

Case 6: Secondary wound site infection

**Figure 6:**
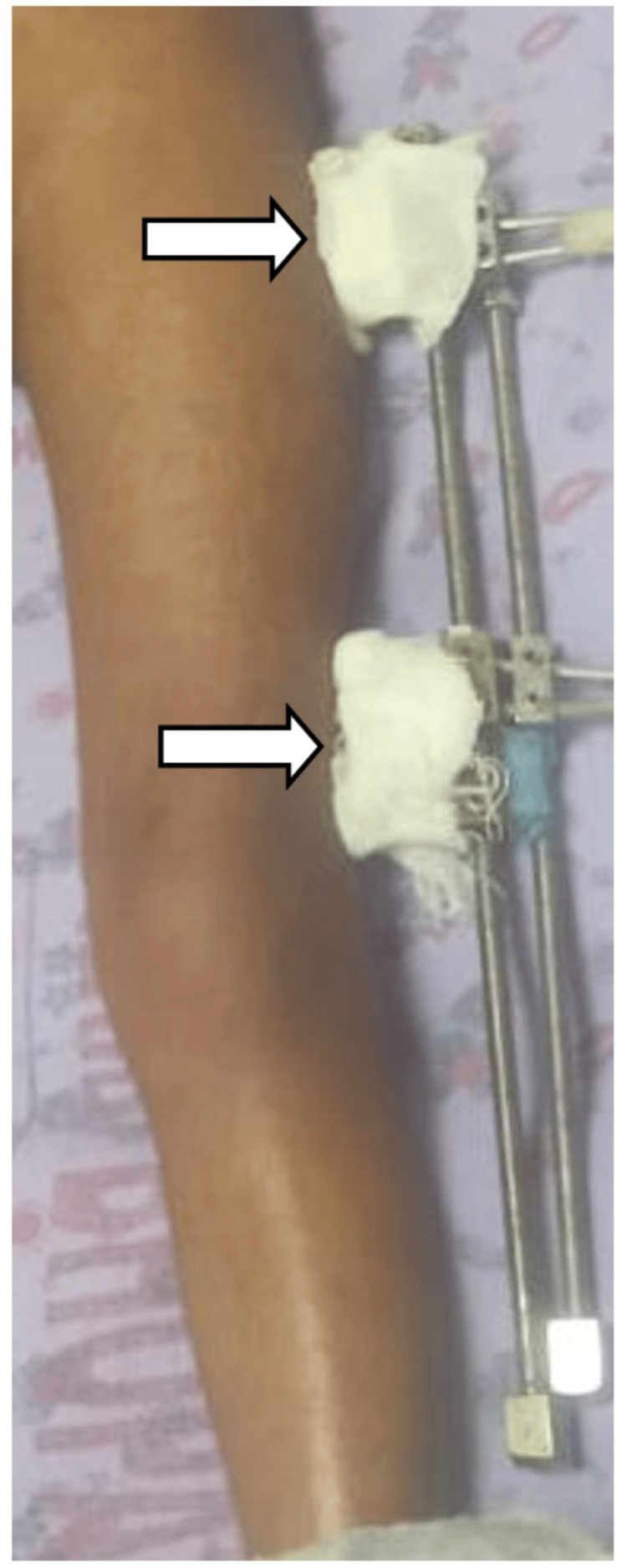
External fixator site of the left leg (white arrows)

A four-year-old malnourished boy, known case of spina bifida occulta, presented in OPD with abdominal pain and whitish urine for one week. The patient had previously undergone femur osteotomy and right pyeloplasty six months ago. Examination revealed undernutrition, pallor, and an external fixator in situ (Figure 6). Urinalysis was positive for leukocyte esterase and nitrite. USG (KUB) showed right bilateral hydronephrosis, suggestive of pelvi-ureteric junction obstruction for which percutaneous nephrostomy was done. During hospitalization, he developed fever spikes and pus discharge at the site of the external fixator. While urine cultures remained sterile, pus from the fixator site grew Bpm. He was continued on iv meropenem for a week, followed by ceftazidime for three weeks, leading to clinical improvement. Further evaluation revealed neurofibromatosis (café-au-lait spots), bilateral Lisch nodules, and a positive family history, confirming neurofibromatosis type 1. He was discharged on oral co-trimoxazole for three months. Patient continued to have repeated UTI for which he had undergone repeat pyeloplasty in the pediatric surgery department. Patient is under follow-up with no repeat cultures positive for Bpm, till September 2025.

Case 7: Brain abscess

**Figure 7:**
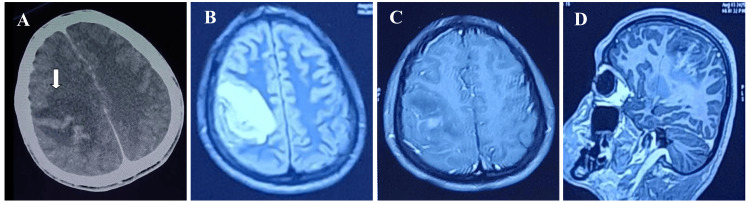
Axial contrast-enhanced computed tomography (CECT) brain (a) showing moderate sized expansile hypodense area (white arrow) in right pre- and post-central gyri, showing gyriform enhancement along with leftward midline shift secondary to mass effect and effacement of sulcal spaces due to diffuse cerebral edema. Axial fluid-attenuated inversion recovery (FLAIR) (b), Contrast-enhanced axial (c), and contrast-enhanced parasagittal section (d) on MRI showing similar findings with well-defined abnormal T2/FLAIR hyperintense area in right pre- and post-central gyrus showing irregular central contrast enhancement suggestive of focal cerebritis with evolving abscess.

A 16-year-old male patient, with no known comorbidities, presented in the emergency with complaints of headache for 20 days, one episode of seizure, and hemiplegia for one day. On examination, the patient was afebrile, and vitals were stable with GCS E4M5V6. On neurological examination, power was 3/5 in the left upper and lower limbs, extensor plantar reflex, and left facial palsy. MRI brain suggested a right fronto-parietal intracranial space-occupying lesion with mass effect. Contrast-enhanced CT brain confirmed the findings with ring enhancement indicating brain abscess (Figure 7A-7D).

The patient suffered from status epilepticus and was admitted to the neurosurgery ICU and started on iv antileptics and piperacillin-tazobactam. Due to deteriorating sensorium, he underwent emergency decompressive craniectomy with evacuation of brain abscess. Samples were sent for aerobic culture, cartridge-based nucleic acid amplification test (CB-NAAT), and histopathological examination. Pus culture grew Bpm, while CB-NAAT was negative for Mycobacterium tuberculosis, and histopathological examination was suggestive of mixed inflammatory infiltrates, macrophages showing hemophagocytosis, with no evidence of granuloma. Antimicrobial therapy was escalated to meropenem as per the susceptibility testing report. Initially, the patient’s sensorium improved and was extubated. However, he later developed recurrent seizures, fever spikes, and septic shock. He was again shifted to ICU, where he suffered cardiac arrest and could not be revived.

## Discussion

We found that over six years, we diagnosed 132 cases of melioidosis, of which seven (5%) were in pediatric and adolescent age groups. The cases presented at our institute had a wide spectrum of clinical manifestations ranging from mild suppurative infection, localized visceral involvement, to an acute fulminant disease leading to mortality in two (28%) patients. It underscores the importance of suspecting Bpm as an etiology in a wide variety of infectious disease syndromes. A similar proportion of involvement in the pediatric population was noted in a longitudinal study from northern Australia, wherein between 1989-2013, of 820 cases, 45 (5%) were in the pediatric age group [8]. In India, Mukhopadhyay et al. from southern India reported 11 (8%) pediatric cases between 2007-2014 [13].

We observed the lung as the most common organ involved in the pediatric population, as three (42%) had features of pneumonia which is the most common presentation in adults [16]. However, involvement of the head and neck region is more commonly reported in pediatric melioidosis [7,8]. In case series from Southeast Asian countries such as Malaysia and Vietnam, melioidosis mainly presents as suppurative parotitis or cervical lymphadenitis [18,19], whereas in Australia, primary cutaneous involvement is predominant [8]. In contrast, in our study, only one (14%) child presented with cervical lymphadenitis, and none had parotitis. Also, two (28%) patients presented with involvement of visceral organs, which is unusual in the pediatric population. As our institute is a tertiary care institute wherein more critically ill or chronic cases with long-standing symptoms present, such trends may be noted.

In adults, diabetes is the most frequent predisposing factor [16]; however, in our study, none of the patients was diabetic, nor had raised sugar levels at the time of clinical presentation. Like adults, we also noted a strong male gender preponderance in diagnosed cases. Moreover, four (57%) children were without any comorbidity. While the poor outcomes are associated with pre-existing comorbidities [7], in our cases, mortality occurred in patients with no known comorbidities.

Melioidosis is increasingly being recognized as an emerging infectious disease in India [20]. Most reported cases have been from coastal states, particularly in southern and eastern India [4]. It is increasingly recognized from other parts of India, including western and northern India [20], with limited documented cases from central India [21]. This study is from the state of Madhya Pradesh, a landlocked state with an agrarian economy located in central India. These seven patients were not geographically related to each other, belonging to six different districts, indicating widespread presentation of the disease in the state.

The combination of moderate to high rainfall and expanding paddy cultivation in recent years in the region may have created favorable environmental conditions for the survival of B. pseudomallei in soil and surface water [22]. All except one case presented during the monsoon and post-monsoon period between July and October which is similar to the trend observed from southern India [3,23]. Four of our patients belonged to the rural socio-economic background, with paddy cultivation being done either in their households or nearby fields; however, we also diagnosed cases in patients living in urban locations. It is quite possible that the presence of the bacterium in household drinking water may either lead to primary or reactivation disease. Recently, there has been a link of melioidosis cases with contaminated water in community and hospital settings as well [24-26]. These findings suggest a shifting epidemiology, highlighting the need for increased clinical awareness and surveillance for melioidosis in traditionally non-endemic, non-coastal regions.

Our study is limited in being a single-center study with a limited number of pediatric cases, which may not fully represent the complete clinical spectrum of melioidosis in this region. In the future, multicentric studies with larger cohorts should be considered for delineating the clinical spectrum. However, the key strength of this study is that it presents pediatric melioidosis cases diagnosed at our center over around six years, providing the longitudinal insight into the clinical spectrum from a region that is traditionally not considered endemic for melioidosis. We also aggressively looked for melioidosis by employing selective culture techniques on pus and respiratory samples to increase the case detection rate.

## Conclusions

To conclude, melioidosis presents with protean clinical presentations in pediatric patients and should be considered as an important differential diagnosis especially in patients with community-acquired pneumonia, visceral abscesses and lymphadenitis. In children, it is present without the traditional risk factors such as diabetes mellitus and chronic alcohol use. If undiagnosed, melioidosis is associated with very high mortality and emphasis on early diagnosis and specific treatment cannot be stressed enough. In our study, the endemicity of the disease in a non-coastal region in central India indicates a shift in epidemiological pattern and highlights the need for increased clinical vigilance. The disease is expanding its geographical footprints in India which could be due to increased recognition, awareness amongst clinicians and improved diagnostics.
